# Comparative Proteomic Profiling Identifies Reciprocal Expression of Mitochondrial Proteins Between White and Gray Matter Lesions From Multiple Sclerosis Brains

**DOI:** 10.3389/fneur.2021.779003

**Published:** 2021-12-24

**Authors:** Nagendra Kumar Rai, Vaibhav Singh, Ling Li, Belinda Willard, Ajai Tripathi, Ranjan Dutta

**Affiliations:** ^1^Department of Neurosciences, Cleveland Clinic, Cleveland, OH, United States; ^2^Proteomic Core Facility, Lerner Research Institute, Cleveland Clinic, Cleveland, OH, United States; ^3^Cleveland Clinic Lerner College of Medicine, Cleveland Clinic, Cleveland, OH, United States

**Keywords:** multiple sclerosis, mass spectrometry, brain tissue, white matter lesion, mitochondria

## Abstract

Multiple sclerosis (MS) is a chronic inflammatory and demyelinating disease of the central nervous system, where ongoing demyelination and remyelination failure are the major factors for progressive neurological disability. In this report, we employed a comprehensive proteomic approach and immunohistochemical validation to gain insight into the pathobiological mechanisms that may be associated with the progressive phase of MS. Isolated proteins from myelinated regions, demyelinated white-matter lesions (WMLs), and gray-matter lesions (GMLs) from well-characterized progressive MS brain tissues were subjected to label-free quantitative mass spectrometry. Using a system-biology approach, we detected increased expression of proteins belonging to mitochondrial electron transport complexes and oxidative phosphorylation pathway in WMLs. Intriguingly, many of these proteins and pathways had opposite expression patterns and were downregulated in GMLs of progressive MS brains. A comparison to the human MitoCarta database mapped the mitochondrial proteins to mitochondrial subunits in both WMLs and GMLs. Taken together, we provide evidence of opposite expression of mitochondrial proteins in response to demyelination of white- and gray-matter regions in progressive MS brain.

## Graphical Abstract

Graphical summary of the main findings of the study. 
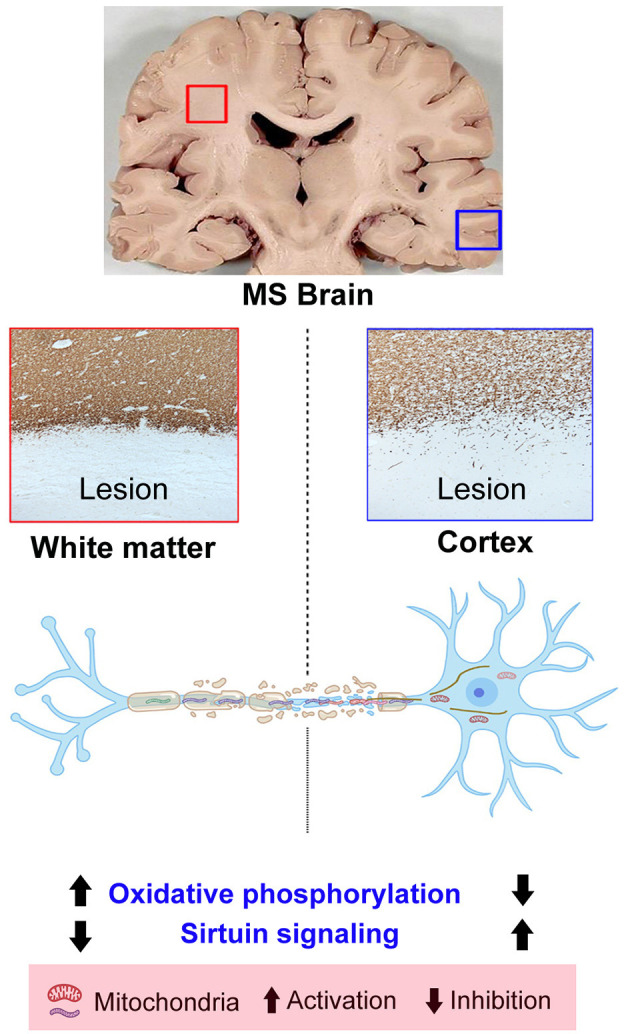

## Introduction

Multiple sclerosis (MS) is a chronic inflammatory demyelinating and neurodegenerative disease of the central nervous system (CNS) with unknown etiology. MS demyelinating lesions are heterogeneous in nature and affect both white- and gray-matter regions in MS brain. Active demyelinating white-matter lesions (WMLs, with abundant microglia/macrophages containing myelin degradation products) are commonly found in relapsing-remitting (RR) patients, while inactive or chronic active demyelinating lesions with a rim of microglia and/or macrophages are more prominent in progressive stages of MS (1, 2). Interestingly, cortical gray matter lesions (GMLs) have also been identified at the earliest stages of MS (3, 4). GMLs are characterized by demyelination, axonal loss, and to a lesser extent blood–brain barrier (BBB) disruption and lymphocyte infiltration (5). Hence, identifying common mechanisms associated with demyelination in WMLs and GMLs will provide a better understanding of the pathogenesis of MS.

With advancements in genomics, transcriptomics, epigenetics, and metabolomics tools, significant efforts have been made to understand MS disease etiology. Recently, proteomic approaches have been used as large-scale and unbiased tools to understand different neurodegenerative diseases (6, 7). Proteomic analysis has a distinct advantage over the transcriptomic assay, which often fails to represent the protein expression level as well as posttranslational modifications (8, 9). Different proteomic approaches have been used to analyze biological samples from patients with MS (10). However, studies analyzing protein profiles of MS demyelinating lesions are scarce. In their report describing WML protein profiles from MS brain, Han and colleagues analyzed proteome from different MS lesion types and found increased levels of proteins involved in the cell cycle, synaptic transmission, cell signaling, whereas the least abundant proteins were associated with cellular metabolism and molecular chaperones (11). In a separate work, utilizing strategies to process formalin-fixed brain tissues from chronic and remyelinating lesions, seven proteins of pathological significance, myelin-associated glycoprotein (MAG), hyaluronan and proteoglycan link protein 2 (HAPLN2), brevican (BCAN), alpha-enolase (ENO1), peroxiredoxin-6 (PRDX6), contactin (CNTN) and glial fibrillary acidic protein (GFAP) were identified (12). EphrinB3 was identified in a proteomic screen of OPC cultures and MS lesions as an inhibitor of OPC differentiation (13). Although these studies provide support for the importance of proteomics-based analysis of MS lesions, direct comparisons between WMLs and GMLs have not been conducted.

**Figure 1:**
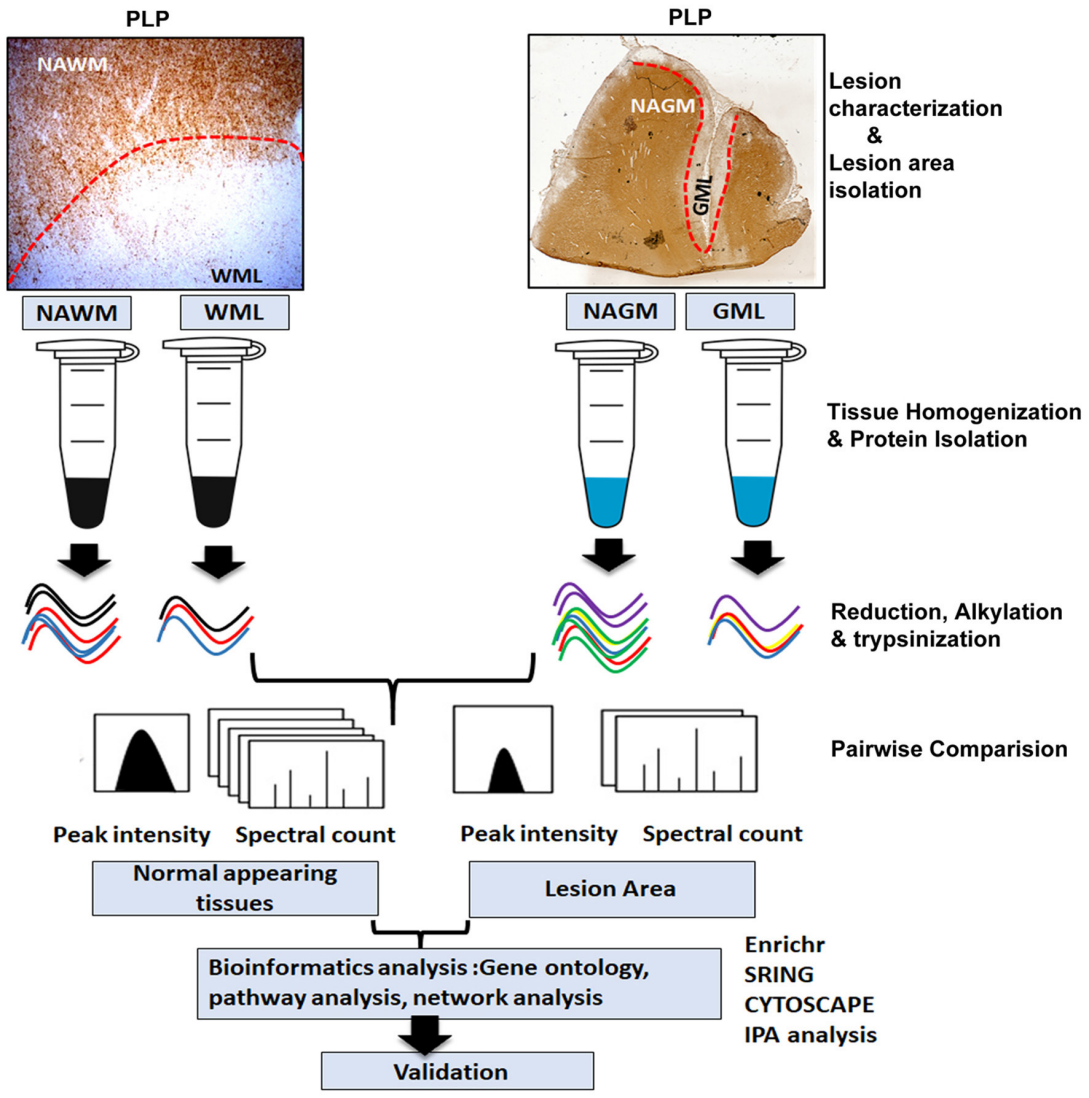
Schematic of proteomics workflow and expression analysis. Study outline including experimental procedures and data acquisition for comparative proteomic analysis of WM and GM normal-appearing and demyelinating lesions (WMLs and GMLs) from progressive MS brain. Peptide and proteins were quantified with label-free algorithms in Max-Quant software. The differentially expressed proteins were further analyzed for gene ontology, protein–protein interaction network analysis, and pathway mapping.

In this work, we used proteomic approaches to identify altered proteins in demyelinating WMLs and GMLs from progressive MS brains compared to myelinated normal-appearing tissues (Figure 1). Label-free quantitative (LFQ) proteomics coupled with liquid chromatography-tandem mass spectrometry (LC-MS/MS) and bioinformatics analysis suggests that a majority of the upregulated proteins in WMLs were associated with mitochondrial respiratory complexes performing biological functions like oxidative phosphorylation. Conversely, the expression levels of many mitochondrial proteins were decreased in GMLs, supporting the concept that the pathobiological-mechanisms arising from myelin loss in WM and GM in MS brains are different.

## Experimental Procedures

### Human Tissue Collection

Deidentified human brain samples from patients with MS were obtained from the rapid autopsy program approved by the Institutional Review Board of Cleveland Clinic (14). Briefly, postmortem brains were sliced (1 cm thick) and then either fixed in 4% paraformaldehyde for morphological studies or rapidly frozen for proteomic studies. Patient demographics are shown in Table 1.

**Table 1:** Demographics used for proteome profiling and immunohistochemistry analysis of the patient with MS.

**Sample ID**	**Sample types**	**Age (Yr)/ sex**	**Postmortem interval (hr)**	**MS types**	**Disease duration (Yr)**	**EDSS score**
**MS samples used for Proteome profiling**						
MS 1	NAWM	75/M	5.0	PPMS	23.7	9
MS 2	NAWM	74/M	9.0	SPMS	35.5	8
MS 3	NAWM	61/F	7.0	PPMS	13.8	7.5
MS 4	NAWM	50/F	9.5	SPMS	17.0	9.5
MS 7	NAWM	65/M	7.0	SPMS	19.4	8
MS 8	NAWM, WML	27/M	8.4	PPMS	1.8	8
MS 5	NAWM, WML	51/F	10.0	SPMS	23.0	7
MS 6	NAWM, WML	61/M	11.0	SPMS	43.0	6.5
MS 9	WML	54/F	8.2	RRMS	15.6	1
MS 10	WML	35/M	9.5	SPMS	21.0	9.5
MS 11	WML	69/F	5.0	PPMS	-	-
MS 12	WML	60/F	7.8	SPMS	29.5	9
MS 13	WML	59/F	5.0	SPMS	37.5	9
MS 14	WML	52/M	17.5	SPMS	12.7	8
MS 15	NAGM	75/M	5.4	PPMS	23.7	9
MS 23	NAGM	65/M	7.0	SPMS	19.4	8
MS 24	NAGM	27/M	8.4	SPMS	1.8	8
MS 25	NAGM	52/M	4.8	SPMS	25.1	9.5
MS 29	NAGM	52/M	17.6	SPMS	12.7	8
MS 30	NAGM	67/F	7.7	SPMS	28.8	6.5
MS 21	NAGM	51/F	10.0	SPMS	23.0	7
MS 16	NAGM, GML	74/M	8.9	SPMS	35.5	8
MS 22	NAGM, GML	61/M	11.0	SPMS	43.0	6.5
MS 17	GML	70/F	5.0	PPMS	17.4	8
MS 18	GML	77/F	5.7	SPMS	54.0	8
MS 19	GML	38/M	12.0	SPMS	1.0	8
MS 20	GML	50/F	9.5	SPMS	17	9.5
MS 26	GML	57/M	9.6	SPMS	27.5	6.5
MS 27	GML	60/F	7.8	SPMS	29.5	9
MS 28	GML	59/F	5.0	SPMS	37.5	9
**MS samples used for immunohistochemical staining**						
MS 31	NAWM, WML	45/M	3	SPMS	36	7
MS 32	NAWM, WML	56/M	3	SPMS	32.7	9.5
MS 33	NAWM, WML, NAGM, GML	59/F	5	SPMS	37.5	9
MS 34	NAGM, GML	70/M	10	PPMS	17.1	6.5
MS 35	NAGM, GML	71/F	5	SPMS	27.1	9.5

*RRMS, Relapsing remitting multiple sclerosis; SPMS, Secondary- progressive multiple sclerosis; PPMS, Primary progressive multiple sclerosis; PMI, Post mortem interval*.

### Proteomic Sample Preparation

Cryostat sections (14 μm) of fresh frozen tissue blocks with myelinated, normal-appearing white matter (NAWM) tissue from eight patients with MS and demyelinated WM (WML *n* = 9 patients) regions were selected based on proteolipid protein (PLP) and major histocompatibility complex (MHC)-class II staining. MHCII staining showed immune cells bordering the WM lesions, suggesting chronic active nature of the WMLs. Additionally tissue from normal-appearing gray matter (NAGM) from nine patients with MS with demyelinated subpial gray matter (GMLs) areas from nine progressive MS brains were also selected (Patient demographics are shown in Table 1). Myelinated and lesioned areas were scored on the frozen block with a scalpel before cutting into 60 μm-thick sections and processed for total lysates preparation, as previously described (15). Briefly, tissue was lysed in RIPA lysis buffer (Thermo Scientific) with tissue homogenizer followed by sonication (Ultrasonic Disruptor Sonifier II, Model W-250/W-450, Bransons Ultrasonics, Danbury, CT, USA) for 1 min at 70% amplitude. Total protein concentration was measured by Pierce^TM^ bicinchoninic acid protein assay kit (Thermo Scientific, Rockford, IL, USA) and stored at −80°C until ready to use. Fifty micrograms of tissue lysate from the respective sample were digested using an in-solution trypsin digestion protocol, as previously described (16), followed by denaturation at 99°C for 5 min, reduction with 5 mM dithiothreitol (DTT) at 60°C for 30 min, and alkylation with 15 mM iodoacetamide in the dark at room temperature for 60 min, respectively. Samples were then incubated for digestion overnight at 37°C by adding trypsin in a 1:50 protease: protein ratio. After digestion, RapiGest was degraded by acidifying to 0.5% (v/v) TFA and incubating for 30 min at 37°C. Samples were desalted through ZipTip C18 and vacuum dried (15).

### LC-MS/MS Measurements

Digested peptides were analyzed on a ThermoFisher Scientific UltiMate 3000 HPLC system (ThermoFisher Scientific, Bremen, Germany) interfaced with a ThermoFisher Scientific Orbitrap Fusion Lumos Tribrid mass spectrometer (Thermo Scientific, Bremen, Germany). Liquid chromatography was performed prior to MS/MS analysis for peptide separation. The HPLC column used is a Dionex 15 cm x 75 μm Acclaim Pepmap C18, 2 μm, 100 Å reversed-phase capillary chromatography column. Five-microliter volumes of the peptide extract were injected, and peptides were eluted from the column by a 90-min acetonitrile/0.1% formic acid gradient at a flow rate of 0.30 μL/min and introduced to the source of the mass spectrometer online. The nanoelectrospray ion source was operated at 2.3 kV. The digest was analyzed using the data-dependent multitask capability of the instrument acquiring full scan mass spectra using a Fourier Transform (FT) orbitrap analyzer to determine peptide molecular weights and collision-induced dissociation (CID) MS/MS product ion spectra with an ion-trap analyzer to determine the amino acid sequence in successive instrument scans. The MS method used in this study was a data-dependent acquisition (DDA) with 3-s duty cycles. It includes one full scan at a resolution of 120,000 followed by as many MS/MS scans as possible on the most abundant ions in that full scan. Dynamic exclusion was enabled with a repeat count of 1, and ions within 10 ppm of the fragmented mass were excluded for 60 s.

### Protein Quantification, Identification, and Data Analysis

The data were analyzed using MaxQuant V1.6.1.0 with the search engine Andromeda, which is integrated with MaxQuant software. The parameters used were default settings for an Orbitrap instrument (17). The database used to search the MS/MS spectra was the Uniprot human protein database containing 25,035 entries with an automatically-generated decoy database (reversed sequences). The search was performed looking for fully tryptic peptides with a maximum of two missed cleavages. Oxidation of methionine and acetylation of protein N-terminus were set as dynamic modifications, and carbamidomethylation of cysteine was set as static modifications. The precursor mass tolerance for these searches was set to 20 ppm, and the fragment ion mass tolerance was set to 0.5 Da. The search was performed including the common contaminant database available in MaxQuant, and these proteins were excluded in the data analysis. A false discovery rate (FDR) was set to 1% for both peptide and protein with a minimum length of seven amino acids, one unique and two total peptides were required for positive identification. Further to analyze biological pathways associated with differentially expressed proteins (DEPs, *p* < 0.05) in WMLs/GMLs, the ingenuity pathway analysis (IPA, Ingenuity Systems, Redwood City, CA, United States) tool was employed to report active and inhibited pathways based on *z*-score and *p* values. The mass spectrometry proteomics data have been deposited to the ProteomeXchange Consortium *via* the PRIDE (18) partner repository with the dataset identifier PXD029285.

### GO Enrichment and Protein–Protein Interaction Analysis

The enrichment analysis of liquid chromatography-mass spectrometry (LC-MS) identified DEPs (*p* < 0.05) was performed by the EnrichR tool (http://amp.pharm.mssm.edu/Enrichr/) (19). Network analysis was performed using STRING to construct a protein–protein interaction (PPI) network of DEPs based upon the known and predicted PPIs with a confidence score of ≥0.7 (20). Furthermore, the PPI network was visualized, and the importance of each protein was analyzed by using Cytoscape software (version 3.7.2) and topological network indices, namely degree, closeness centrality, centrality, and the number of direct edges for each protein were calculated by using Cytoscape plugin-Network analyzer. MCODE Cytoscape plug-in was used to identify highly interconnected clusters in the PPI network (21).

### Immunohistochemistry

All immunostaining was performed on 30 μm free-floating sections of fixed tissues or 5–7 μm frozen tissue sections from snap-frozen tissue blocks in 5 additional MS brains (Table 1). Free-floating sections (30 μm thick) were washed in phosphate-buffered saline (PBS), processed for heat-induced antigen retrieval in citrate buffer (10 mM, pH- 6), and blocked in 5% normal goat serum followed by incubation in primary antibodies for rat anti-proteolipid protein 1 (PLP1 (1:250); hybridoma, a gift from W. Macklin, Department of Cell and Developmental Biology, University of Colorado School of Medicine, Aurora, CO), mouse anti-major histocompatibility complex class II (MHCII (1:250), M0775; Dako, Agilent Technologies, Santa Clara, CA, USA), rabbit anti-Ermin (ERMN, (1:500), NBP1-84801; NovusBiologicals, Centennial, CO), rabbit anti-carnosine dipeptidase 1(CNDP1 (1:50), PA5-26174; Invitrogen Inc., USA), and rabbit anti-ectonucleotide pyrophosphatase/phosphodiesterase 2 (ENPP2 (1:50), PA5-12478; Invitrogen Inc. USA), as previously described (22). Frozen sections were fixed in cold methanol and blocked in 5% normal goat serum followed by incubation in PLP1 (1:100), MHCII (1:250), rabbit anti-voltage-dependent anion channel (VDAC (1:250), ab14734; Abcam Inc., USA), rabbit anti-ubiquinone oxidoreductase subunit A5 (NDUFA5 (1:500), PA5-22143; Invitrogen Inc., USA) and rabbit anti-ATP synthase membrane subunit G (ATP5L (1:200), PA5-60783; Invitrogen Inc., USA). After PBS washing, sections were incubated with corresponding biotinylated secondary antibodies (Vector Laboratories, Burlingame, CA), avidin–biotin (ABC), and diaminobenzidine (DAB) treatment. Digital bright-field images were obtained using a Leica DM5500 microscope. Sections for confocal fluorescence microscopy were pretreated as described above, incubated (2–3 days) with primary antibodies of mouse anti-Olig2 (1:250, MABN50; Millipore Inc., USA) against rabbit antibodies for identified downregulated OLs proteins ERMN(1:500), CNDP1 (1:50), ENPP2 (1:50), on sections previously costained for MHC II and PLP, and then incubated with secondary antibodies conjugated to either Alexa Fluor 594 or Alexa Fluor 488 (Thermo Fisher Scientific Inc., USA) for 1–2 h.

### Experimental Design and Statistical Rationale

A Wilcoxon test (to compare non-normally distributed abundance) was applied for testing the differences in the abundance of proteins between samples of WML/NAWM, and GML/NAGM groups. Relative quantitative differences in protein abundance between samples were calculated as log2 ratios between mean LFQ abundances of both groups *with p* < 0.05 considered statistically significant.

## Results

### Proteomic Profile of Demyelinating WMLs

**Figure 2:**
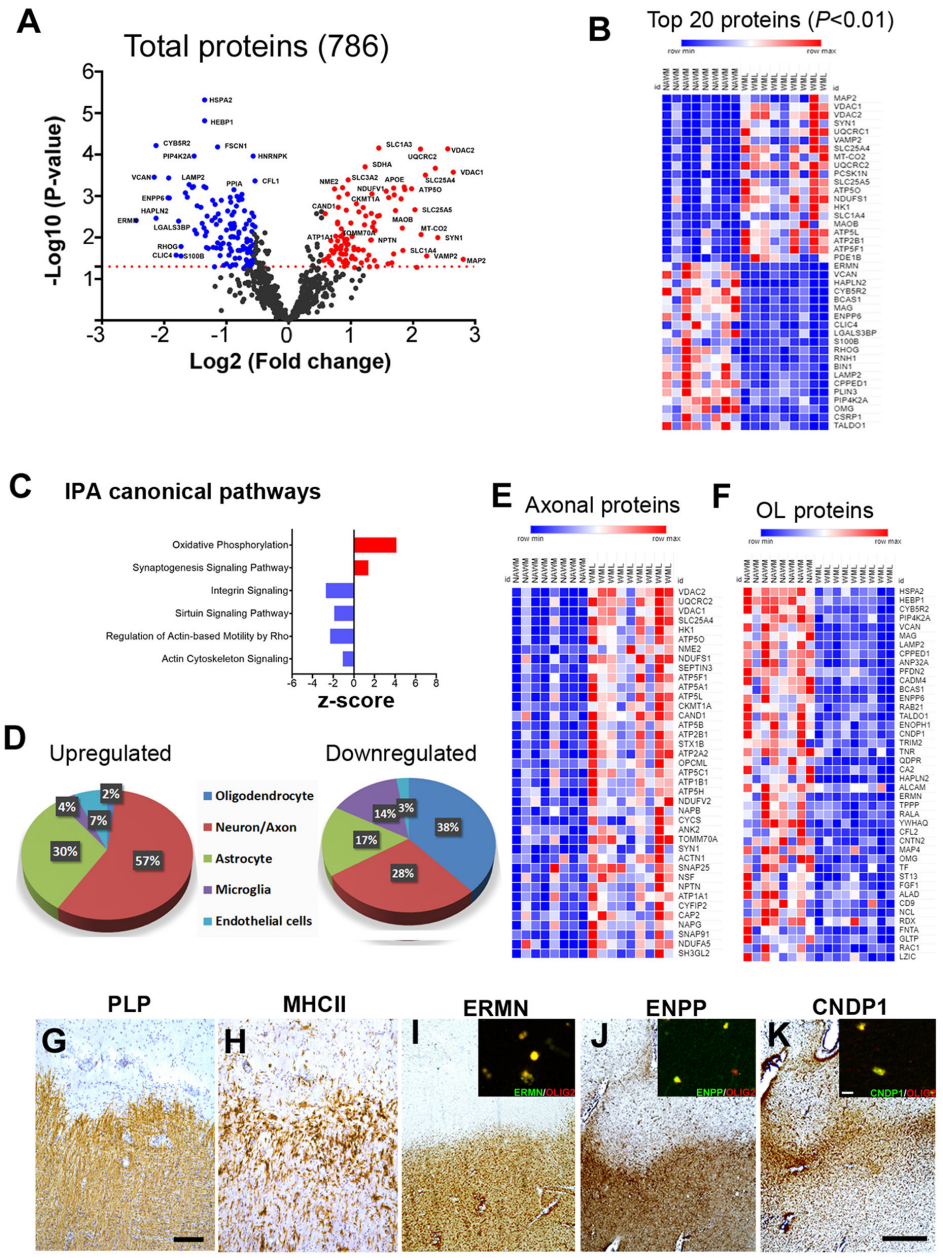
MS brain proteome analysis and validation in WMLs. **(A)** Volcano plot displaying the distribution of all identified proteins (*n* = 786) with relative protein abundance (log2 fold change) plotted against levels of significance (-Log_10_ *p*-value). 105 proteins were significantly upregulated (red circle) while 119 were downregulated (blue circle) in WMLs. **(B)** Mean LFQ intensity-based heatmap showing the top 40 dysregulated proteins (20 upregulated and 20 downregulated) in MS WMLs. The color range of LFQ intensity extends from low (dark blue) to high (dark red). **(C)** IPA canonical pathways found to be activated (red) and inhibited (blue) among significant DEPs in WMLs. **(D)** Cell-specific expression of upregulated and downregulated proteins in WMLs. **(E,F)** Mean LFQ intensity-based heat map showing axonal **(E)** and oligodendrocytes (OL) specific proteins **(F)** in WMLs. **(G–K)** IHC images showing Di-amino benzidine (DAB) images of PLP **(G)**, MHCII **(H)** ERMN **(I)**, CNDP1 **(J)**, and ENPP2 **(K)** in the chronic WMLs from progressive MS brain. Sections **(G,H)** were counterstained with Haematoxylin. The inset shows immunofluorescence images of Olig2 co-localization with ERMN **(I)**, ENPP **(J)**, CNDP1 **(K)**. Scale bar: **(G,H)**, 200μm; **(I–K)**, 500 μm; inset, **(I–K)**, 10 μm.

To identify WML proteomes from progressive MS brains, total protein was isolated and processed for LFQ-MS measurements (Patient details in Table 1). The amount of digested protein samples quantified with LC-MS/MS did not show any significant difference (*p* = 0.54) between WML and NAWM samples (data not shown). We identified a total of 41,023 MS/MS spectral counts from NAWM and 59,218 from WML samples, respectively. Following the database search and data filtering, a total of 9,588 peptides corresponding to 2,045 proteins were identified from the tryptic digested samples and mapped to 786 unique gene symbols across the 17 samples (NAWM-8 and WML-9) (Supplementary File 1). Comparing the abundance of identified proteins (*n* = 786) from WML and NAWM samples, 224 proteins were found to be significantly changed (*p* < 0.05), of which 105 proteins were significantly upregulated and 119 were significantly downregulated in WML (Figure 2A; top 40 proteins are shown in Figure 2B). As expected, myelin proteins (log2 fold change, MAG = −1.93, ERMN = −2.45, BCAS1 = −1.94, OMG = −1.51, VCAN = −2.16) were found to be downregulated in demyelinated WMLs of progressive MS brains (Supplementary File 1). Analyzing the pathways associated with significantly dysregulated DEPs, IPA analysis showed activation of oxidative phosphorylation (z score = 4.123, *p* < 0.00001) and synaptogenesis signaling pathway (z score = 1.4, *p* < 0.00001) in WMLs (23), whereas pathways like sirtuin signaling pathway (z score = −1.89, *p* < 0.00001), regulation of actin-based motility by Rho (z score = −2.3, *p* < 0.00001), and actin cytoskeleton signaling (z score = −1.07, *p* < 0.00001) were found to be inhibited in WMLs (Figure 2C, Supplementary File 2). Interestingly, many of these pathways have been previously identified by transcriptome profiling of WMLs (24, 25). Further, to identify the cellular source of DEPs in WMLs, we queried significantly DEP to the CNS cell-specific database (26). The bulk of upregulated proteins were mapped to neuronal/axonal origin (57%) and astrocytes (30%) (Figures 2D,E, Supplementary Table 1). On the contrary, a majority of the downregulated proteins (38%) like CNDP1, BCAS-1, HAPLN2, ENPP2, VCAN, ERMN, MAG, and OMG were associated with oligodendrocyte (OL) lineage cells (27) (Figures 2D,F). In line with the decrease in OL-specific proteins in WMLs, (28, 29), fluorescent colabeling of Olig 2 (pan-oligodendroglial marker) with ERMN, ENPP, and CNDP1 showed OL lineage-specific expression of these proteins in MS brains (Figures 2G–K). Taken together, proteomic analysis confirmed a decrease in OL-specific proteins and showed the involvement of pathways associated with cellular energetics and axonal transport impairment (30) due to demyelination in progressive MS brain.

### WML Proteomes Show Dysregulation of the Mitochondrial Complex and Cytoskeletal Proteins

**Figure 3:**
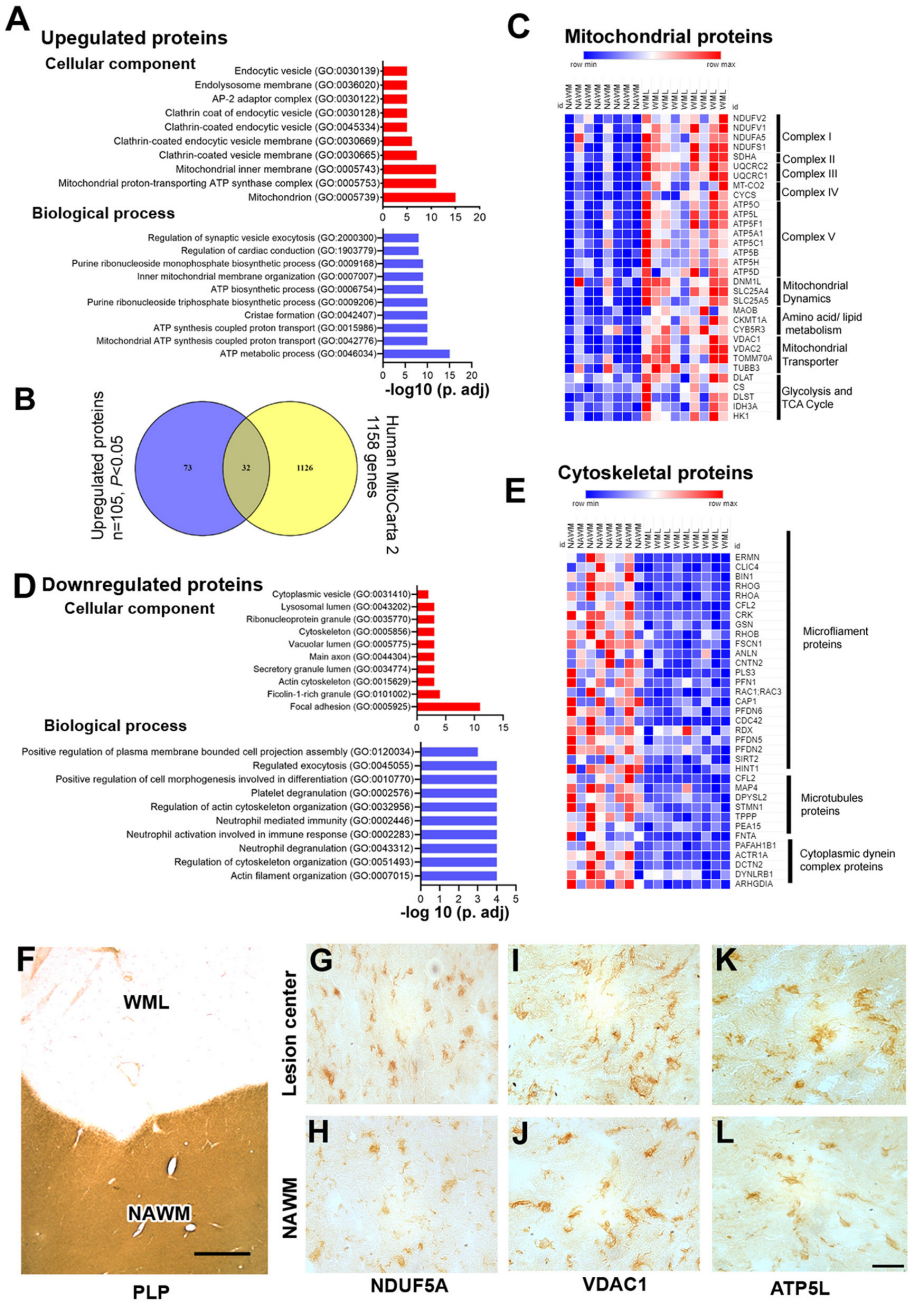
Gene enrichment analysis and validation of WML DEPs. **(A)** Bar graphs showing cellular component (red) and biological process (blue) enrichment of upregulated WML proteomes. **(B)** Venn diagram showing a comparison between mitochondrial genes from the mitocarta 2.0 dataset and upregulated proteins identified in our study. **(C)** LFQ-based heatmap showing upregulated mitochondrial protein distribution across different mitochondrial complexes. **(D)** Bar graphs showing cellular component (red) and biological process (blue) enrichment of downregulated WML proteomes. **(E)** LFQ-based heat map showing downregulated proteins related to microfilament proteins, microtubule proteins, and cytoplasmic dynein dynactin complex proteins. **(F–L)**. Representative IHC images showing cellular localization of VDAC1, NDUFA5, and ATP5L **(G–L)** WMLs and NAWM from progressive MS brains. Scale bar- 500μm **(F)** and 20μm **(G–L)**.

As mitochondrial dysfunction and oxidative phosphorylation have been associated with MS (31), we elected to categorize proteins associated with cellular components and biological processes by enrichment analysis (32). Gene ontology (GO) analysis of upregulated proteins showed that mitochondria (GO: 0005739), mitochondrial proton-transporting ATP synthase complex (GO: 0005753), mitochondrial inner membrane (GO: 0005743), clathrin-coated vesicle membrane (GO: 0030665), endolysosome membrane (GO: 0036020), and endocytic vesicle (GO: 0030139) were enriched as cellular components (Figure 3A, Supplementary File 3). Similarly, biological process analysis showed that upregulated proteins were related to the ATP metabolic process (GO: 0046034), mitochondrial ATP synthesis coupled proton transport (GO: 0042776), cristae formation (GO: 0042407), ATP biosynthetic process (GO: 0006754), and inner mitochondrial membrane organization (GO: 0007007) (Figure 3A, Supplementary File 3). As the majority of upregulated proteins were connected with mitochondrial components/function, we sought to identify different mitochondrial proteins upregulated in WMLs. To achieve this objective, we mapped WML upregulated proteins (*p* < 0.05) into the human mitochondria database (Human MitoCarta2.0) (33). Of the 1,158 assigned mitochondrial proteins, 32 were common to upregulated proteins identified in WMLs (Figure 3B) and belonged to mitochondrial complexes and membrane components like mitochondrial electron transport chain complex I (*n* = 4), complex II (*n* = 1), complex III (*n* = 2), complex IV (*n* = 2), complex V (*n* = 8), mitochondrial dynamics (*n* = 2), amino acid and lipid metabolism (*n* = 3), mitochondrial transporters (*n* = 4), and five proteins were associated with glycolysis and enzymes for the TCA cycle (Figure 3C). We also validated proteomic results of three mitochondrial proteins, voltage-dependent anion channel 1 (VDAC1, a mitochondria specific marker), ubiquinone oxidoreductase subunit A5 (NDUFA5, a marker for mitochondrial respiratory chain complex I), and ATP synthase subunit G (ATP5L, a marker of mitochondrial respiratory chain complex V) in WMLs with immunochemical (IHC) staining and found increased cellular expression of the respective proteins in the lesion centers of WMLs in progressive MS brains (Figures 3F–L) associated with cells exhibiting ramified microglia-like morphology. The increased expression of mitochondrial proteins suggests a possible involvement of a putative compensatory mechanism to cope with the increased energy demand and to restore axonal and cellular function in demyelinated WMLs.

Similarly, we applied GO term enrichment analysis to identify cellular and biological components of the significantly downregulated proteins (*P* < 0.05). We found cell component categories of focal adhesion (GO: 0005925), actin cytoskeleton and (GO: 0015629), cytoskeleton (GO: 0005856) as significantly enriched terms (Figure 3D, Supplementary File 3), whereas actin filament organization (GO: 0007015), regulation of cytoskeleton organization (GO: 0051493), and neutrophil degranulation (GO: 0043312) were identified as the prominent biological processes (Figure 3D, Supplementary File 3). Moreover, organizing the downregulated proteins based on cellular component, the majority of proteins could be categorized into three major components; microfilament proteins (ERMN, CLIC4, BIN1, RHOG, RHOA, CFL2, CRK, GSN, RHOB, FSCN1, ANLN, CNTN2, PLS3, PFN1, RAC1, CAP1, PFDN6, CDC42, RDX, PFDN5, PFDN2, SIRT2, and HINT1), microtubules proteins (MAP4, DPYSL2, STMN1, TPPP, PEA15, and FNTA), and cytoplasmic dynein complex proteins (PAFAH1B1, ACTR1A, DCTN2, DYNLRB1, and ARHGDIA) (Figure 3E). These results further support previous studies that found downregulation of cytoplasmic dynein in demyelinating lesions of MS brains (34). Thus, reciprocal expression of mitochondrial proteins and cytoskeletal complex proteins suggest inhibition of mitochondrial motility and associated disruption of axonal–glial interaction in progressive MS brains.

### Protein–Protein Interactions in WML DEPs Highlight Mitochondrial Complex Proteins and RHO Signaling

**Figure 4:**
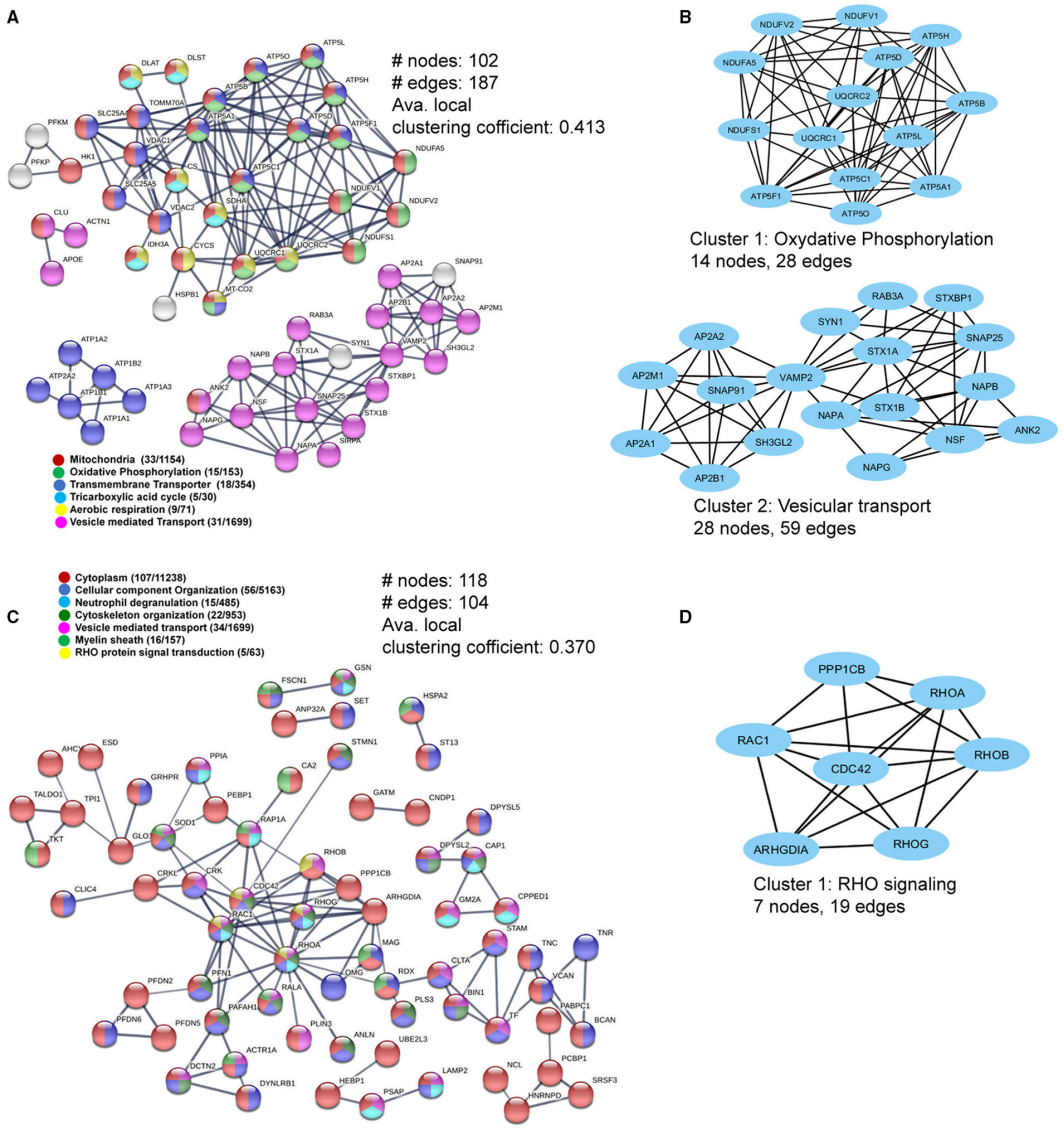
Pathway and interaction network analyses of the differentially-expressed WML proteins. **(A)** PPI network focusing on upregulated mitochondria-related proteins. **(B)** MCODE analyses showing two highly connected module subnetworks (Oxidative phosphorylation and vesicular transport) among the upregulated proteins. **(C)** The protein–protein interaction network for downregulated WML proteins. **(D)** MCODE analysis showing protein module related to RHO signaling among downregulated proteins.

As mitochondrial and cytoskeletal protein complexes were found to be dysregulated in WMLs, we further probed the interactions between these proteins using protein–protein interaction (PPI) network analysis (20) to understand how these proteins are interacting as a complex. PPI network of the upregulated proteins comprised of 102 nodes and 187 edges (confidence score = 0.7; average node degree = 3.67, and average local clustering coefficient = 0.413) (Figure 4A). Average node degree demonstrates average number of interactions for all nodes in the network, and the average clustering coefficient denotes network connectivity of all immediate neighbors of a particular node. The overall interaction map of the upregulated proteins showed proteins belonging to different cellular components like mitochondria (*n* = 33/1154, red), oxidative phosphorylation (*n* = 15/153, green), mitochondrial transmembrane transporters (*n* = 18/354), tricarboxylic cycle (*n* = 5/30, sky blue), aerobic respiration (*n* = 9/71, yellow), vesicle-mediated transport (*n* = 31/1699, violet), respectively (Figure 4A). Further analysis using MCODE-based cluster analysis (21) yielded 2 prominent clusters (highly interconnected regions in a network) that belonged to oxidative phosphorylation (14 nodes, 28 edges) and vesicular transport (28 nodes, 59 edges) (Figure 4B).

Similarly, PPI analysis of the downregulated proteins yielded 118 nodes and 104 edges (confidence score = 0.7; average node degree = 1.76, and average local clustering coefficient = 0.370), smaller than the upregulated proteins, indicating overall lesser interactions among downregulated proteins. The major PPI nodes belonged to cytoplasm (107/11238, Red), regulation of cellular component organization (*n* = 56/5163), blue), neutrophil degranulation (15/485, sky blue), cytoskeleton organization (22/953, dark green), vesicle-mediated transport (34/1699, violet), myelin sheath (16/157, green), and RHO protein signal transduction (*n* = 5/63, yellow), respectively (Figure 4C). The significant cluster (using MCODE) was found to be associated with RHO signaling (7 nodes, 19 edges), (Figure 4D). Thus, the PPI network analysis of DEPs revealed extensive interactions between the mitochondrial proteins and axonal cytoskeletal disorganization, as detected previously (Figures 3C,E).

### Reciprocal Mitochondrial Proteins Expression in Cortical Demyelinating Lesions

**Figure 5:**
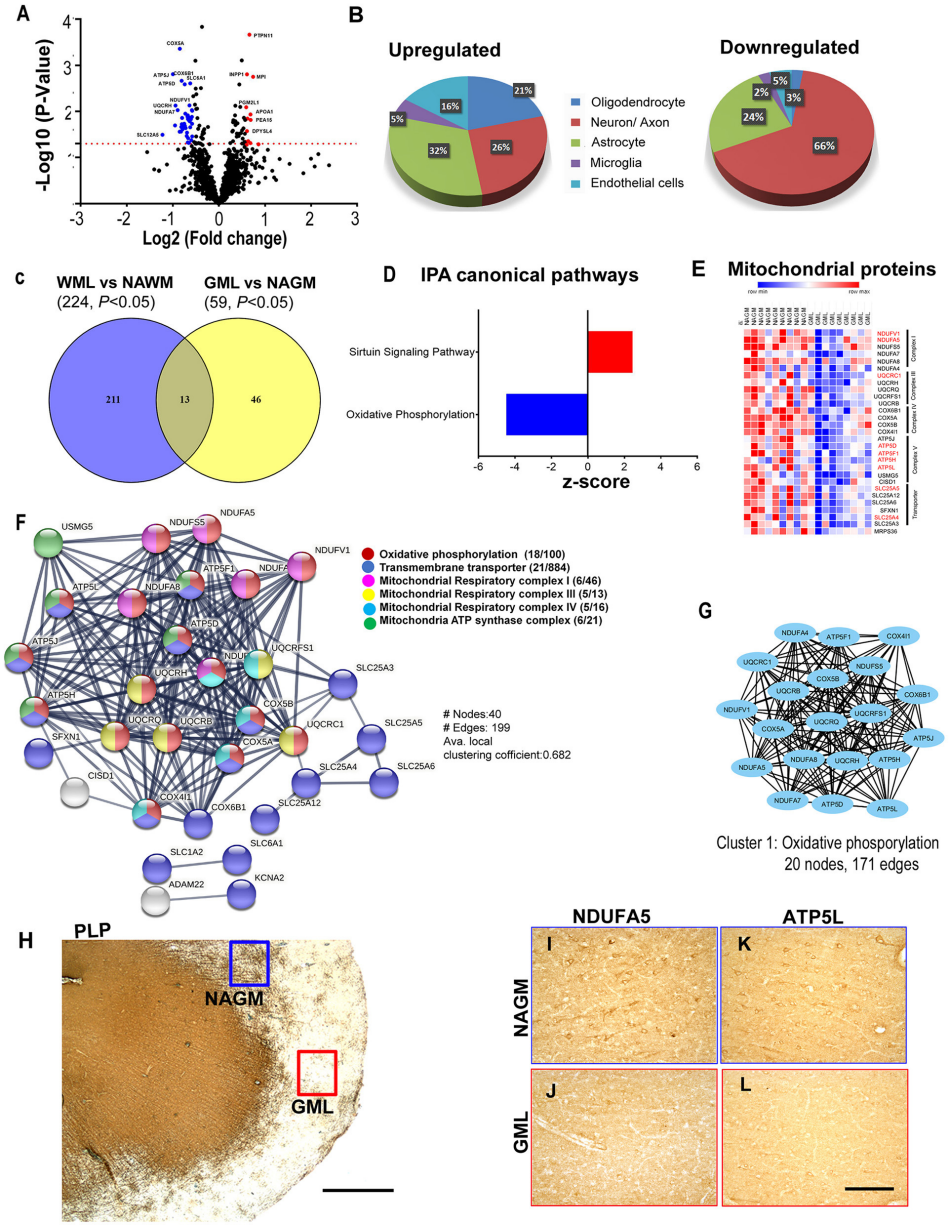
Cortical GML proteome profiling. **(A)** Volcano plot displaying the distribution of all proteins (*n* = 1182) with relative protein abundance (Log2 fold change) plotted against significance level (-Log_10_ *P*-Value). Significantly upregulated (red circle) as well as downregulated (blue circle) proteins in GMLs are also indicated. **(B)** Cellular identity of the significantly dysregulated GML proteins (upregulated and downregulated). **(C)** Venn diagram comparison of DEPs (*p* < 0.05) between WMLs and GMLs. **(D)** IPA canonical pathways, activated (red) and inhibited (blue), associated with significantly dysregulated proteins in GMLs. **(E)** LFQ-based heat map of mitochondria-specific downregulated proteins in GMLs. Proteins that are common and have opposite expressions in WML proteomic data are highlighted in red. **(F)** PPI network analysis of downregulated proteins showing enrichment of mitochondria-related pathways. **(G)** MCODE analysis revealed a highly interconnected sub-network associated with oxidative phosphorylation. **(H–L)** PLP- stained image of **(H)** sub-pial lesion. IHC images showing reduced expression of NDUFA5 **(I,J)** and ATP5L **(K,L)** in demyelinated regions (red box) compared to NAGM (blue box). Scale bar- 500μm **(H)** and 100μm **(I–L)**.

As myelin loss has been observed across CNS tissue including cortical gray matter areas in MS brains (35), we sought to identify dysregulated proteins and associated pathways in demyelinating subpial cortical lesions and to find out whether pathways that are impaired in WMLs are also impacted in GMLs. Hence, total protein was isolated from well-characterized myelinated (NAGM, *n* = 9) and demyelinated (GML, *n* = 9) cortical tissues from age/sex-matched progressive MS brains (Table 1). Similar to WMLs, a total of 85,487 MS/MS spectral counts from NAGM and 81,030 from GML samples were identified. Following the database searching and data filtering, we found a total of 1,182 proteins, 19 significantly upregulated and 40 downregulated proteins, respectively, in GMLs (*P* < 0.05, Figure 5A, Supplementary File 4). We further evaluated for possible CNS cell-specific expression sources and observed that the majority of upregulated proteins (Figure 5B) were associated with astrocytes (32%), whereas more than half (66%) of the significantly downregulated proteins were expressed by neurons (Supplementary Table 2).

While analyzing common proteins expressed during demyelination in WMLs and GMLs, we found 13 similar proteins between the two lesion types (Figure 5C, Table 2). Identifying affected pathways associated with significantly dysregulated GML proteomes, IPA canonical pathway analysis showed possible activation of sirtuin signaling pathway (z score = 2.4, *P* < 0.00001), whereas oxidative phosphorylation (z score = −4.4, *P* < 0.00001) was found to be inhibited in cortical demyelinating lesions of progressive MS brain (Figure 5D, Supplementary File 5). To our surprise, two pathways, the sirtuin signaling pathway and the oxidative phosphorylation had opposite activation patterns in GMLs compared to WMLs (Figure 2C), indicating differential roles/impacts of these pathways in gray and white matter tissues during demyelination. We next compared GML DEPs (59 proteins) with the existing database of human mitochondrial proteins (33) and found 31 common mitochondrial proteins (2 upregulated and 29 downregulated), (Figure 5E), belonging to four mitochondrial complexes (complex I, III, IV, and V). Interestingly, nine significantly downregulated mitochondrial proteins (NDUFA5, NDUFV1, UQCRC1, ATP5F1, ATP5H, ATP5L. ATP5D, SLC25A4, and SLC25A5) had inverse expression profiles in WMLs compared with those in GMLs (Figure 5E).

**Table 2:** List of common significantly differentially expressed proteins between white matter and gray matter proteomes.

**Protein names**	**Gene symbol**	**WML**		**GML**	
		**log2 (FC)**	**-Log_**10**_(P)**	**log2(FC)**	**-Log_**10**_(P)**
ADP/ATP translocase 1	SLC25A4	2.2	3.5	−1	1.6
ADP/ATP translocase 2	SLC25A5	2	2.7	−1	1.3
ATP synthase F(0) complex subunit B1, mitochondrial	ATP5F1	2	3	−1	1.7
NADH dehydrogenase [ubiquinone] 1 alpha subcomplex subunit 5	NDUFA5	1.1	1.7	−1	1.8
Excitatory amino acid transporter 2	SLC1A2	1.7	1.4	−1	1.8
NADH dehydrogenase [ubiquinone] flavoprotein 1, mitochondrial	NDUFV1	1.3	3.1	−1	2.1
ATP synthase subunit d, mitochondrial	ATP5H	1.2	2.3	−1	1.3
Cytochrome b-c1 complex subunit 1, mitochondrial	UQCRC1	2	3.7	−1	1.9
ATP synthase subunit g, mitochondrial	ATP5L	2	2.9	−1	1.7
ATP synthase subunit delta, mitochondrial	ATP5D	1	1.8	−1	2.7
Sodium/potassium-transporting ATPase subunit beta-2	ATP1B2	1	1.7	−1	1.6
Endonuclease domain-containing 1 protein	ENDOD1	−1	1.3	−1	1.4
Astrocytic phosphoprotein PEA-15	PEA15	−1	1.5	1	1.8

Further, GML proteome GO term analysis by EnrichR showed enrichment of GO categories related to focal adhesion (GO: 0005925), secretory granule lumen (GO: 0034774), and cytoplasmic vesicle lumen (GO: 0060205) within upregulated proteins, whereas mitochondrial components (GO: 0005743, GO: 0005739, GO: 0005753, GO: 0005750, GO: 0005751, GO: 0005747, and GO: 0000276), mitochondrial ATP synthesis coupled electron transport (GO: 0042775), respiratory electron transport chain (GO: 0022904), and oxidative phosphorylation (GO: 0006119) were among the downregulated proteins (1, 36) (Supplementary File 6). In addition, PPI network analysis of 19 upregulated GML DEPs did not show any specific interactions; however, analyzing PPI among 40 significantly downregulated proteins showed a network comprised of 40 nodes and 199 edges (Avg. local clustering coefficient: 0.68) (Figure 5F). Likewise, MCODE-based cluster network analysis revealed a cluster associated with oxidative phosphorylation having 20 nodes and 171 edges (Figure 5G). We also validated the proteome findings of two selected mitochondrial complex (complex I and V) proteins, NDUFA5, and ATP5L (having opposite expression patterns in GMLs and WMLs) by IHC staining. In accordance with the proteomic results, we observed reduced expression of NDUFA5 and ATP5L in the lesion area compared with NAGM (Figures 5H–L). Thus, findings from cortical GML proteome analysis indicate impairment of mitochondrial function, possibly in neurons (37), during cortical demyelination in MS brains, and the failure to supplement required energy for proper neuronal activity, thus leading to neurodegeneration in progressive MS and associated neurological disability.

## Discussion

Despite decades of research, the heterogeneous nature and unknown etiology of MS have made it hard to find successful therapeutics. Using label-free quantitative LC-MS/MS proteomics, we identified 224 DEPs in WMLs and 59 proteins in GMLs from progressive MS brains (*p* < 0.05). Associated pathway and protein coexpression network analysis of significantly dysregulated proteins identified reciprocal changes in pathways like oxidative phosphorylation and the sirtuin signaling pathway in WMLs and GMLs. Further, PPI network analysis showed that increased activity of oxidative phosphorylation pathways in WMLs was due to increased expression of proteins belonging to all five mitochondrial complexes (I–V). In addition, decreased expression of proteins in WMLs were related to Rho-signaling and cytoskeletal organization, specifically associated with retrograde transport of mitochondria in WMLs (38). Conversely, we identified decreased expression of the four mitochondrial complexes and the oxidative phosphorylation pathway in GMLs. Taken together, these results provide clues as to the differential mitochondrial responses following demyelination of WMLs and GMLs in progressive MS.

Previous studies have applied proteomic approaches to identify dysregulated proteins in different lesion types (11–13). To reduce heterogeneity between MS cases, comparative proteome profiles were generated using normal tissues surrounding lesion areas from age- and sex-matched progressive cases with MS. Compared to previous studies (11–13), using modern proteomic methods, we identified 224 proteins significantly altered in WMLs compared with only 59 proteins in GMLs. Several conditions like cellular composition, extent of inflammation, and extent of OPCs/OLs could be responsible for these changes (2, 39). It is interesting to note, however, that, unlike rodent CNS, demyelinated lesions in the gray matter of MS brains contain more OPC and OLs, and remyelination is more robust than in WM lesions (40, 41). Our findings are however in line with previous findings in MS lesions, showing greater loss of myelin proteins (38%) in WMLs compared with GMLs (5%) (41).

We applied a system biology approach to identify pathways and protein interactions among significantly dysregulated proteins in both WMLs and GMLs. To our surprise, we noticed two common pathways (oxidative phosphorylation and the sirtuin signaling pathway) having contrasting activity between WMLs and GMLs. In the healthy CNS, mitochondria participate in essential processes such as energy metabolism, calcium homeostasis, and apoptosis (42, 43). Under normal conditions, there is a precise coordination of mitochondrial dynamics between white matter and gray matter, where mitochondria generated in the neuronal cell body are transported to replenish the damaged mitochondria and the damaged mitochondria are transported back to the cell body for degradation (44). It is important to note that increased mitochondrial content, distribution, and activity in the demyelinated axons (WMLs) have been described as a potent adaptive response to increased energy demand [termed as the axonal response of mitochondria to demyelination (ARMD)], and hence might be associated with the upregulated expression of mitochondrial proteins in WMLs (45, 46). On the contrary, reduced cortical mitochondrial proteins expression suggest an apparent neuronal dysfunction/loss, and neuronal mitochondrial defects due to DNA deletion as well as decreased nuclear DNA-encoded transcripts of mitochondrial respiratory chain complexes (23, 47).

Based upon our results, increased synaptogenesis signaling in WMLs (increased expression of synapse-specific proteins) could be a result of a compensatory mechanism against ongoing demyelination in electrically-active axons (48). We also detected reciprocal expression of the sirtuin signaling pathway between MS WMLs and GMLs. Although sirtuins have been involved in immune activity and metabolic regulations, SIRT1, a NAD-dependent protein deacetylase, has been implicated in the pathogenesis of various neurodegenerative diseases including MS (24, 49, 50). Activation of this pathway may also be associated with localization of SIRT3, SIRT4, and SIRT7 in mitochondria (51), which is already affected due to ongoing demyelination, and thus, leads to differential activation in WMLs and GMLs in progressive MS brains, respectively.

Our proteomic data also revealed a significant decrease in the abundance of RHO GTPase signaling proteins such as RHOA, CDC42, RAC1, RHOB, RHOG, PAFAH1B1, and ARHGDIA. Our results are in agreement with previous reports showing an absence of RHOA+ cells in the demyelinating lesion core in MS brain tissues (52), possibly associated with tapering lesions in the inflammatory environment during the progressive stage of MS disease. RHOA function has been associated with the regulation of actin cytoskeleton organization and is essential for the regulation of cell shapes, polarity, motility, and adhesion (53). Inhibition of RHOA GTPase has been associated with the suppression of leukocyte infiltration into the CNS, neutrophil degranulation, reduction in axonal damage and regeneration, and attenuation in the clinical signs of experimental autoimmune encephalomyelitis (54–58).

Cytoskeleton and axonal transport proteins are required for the mitochondrial transport to overcome the ATP demand following demyelination in axons (59). Defects in the axonal transport system have been implicated in demyelinating diseases like MS and many other neurodegenerative disorders (30, 60, 61). As microfilament, microtubule, and retrograde axonal transport proteins (DYNLRB1, ARHGDIA, ACTR1A, DCTN2, and PAFAH1B1) were downregulated in WMLs, defects in cytoplasmic dynein complex proteins could cause specific abnormalities in mitochondrial transport toward the cell body and could lead to mitochondrial accumulation at the demyelinating lesions, as detected in our proteomic results (62, 63). This accumulation of mitochondrial proteins was specific to WMLs, as we found significant downregulation of mitochondrial proteins in GMLs. Collectively, these data provide evidence of increased accumulation of mitochondria due to the lack of retrograde transport in WMLs, whereas decreased levels in GMLs are possibly associated with mitochondrial DNA deletion and related transcription impairment in MS brains.

In summary, our proteomic findings provide evidence strongly supporting differential mitochondrial dynamics in demyelinating lesions of white matter and cortical gray matter regions from progressive MS brain. Proteins related to oxidative phosphorylation and sirtuin signaling pathways highlight the presence of common pathways between two lesion types, despite differential activation. Conversely, downregulation of microfilament/cytoskeletal proteins in WMLs suggests defective recycling of mitochondria due to impaired retrograde axonal transport. Together, these results establish the foundation for future studies to explore the option to target dysregulated proteins/pathways to promote remyelination and neuroprotection for progressive MS disease (Graphical Abstract).

## Data Availability Statement

The datasets presented in this study can be found in online repositories. The names of the repository/repositories and accession number(s) can be found: PRIDE database, PXD029285.

## Author Contributions

NR, VS, AT, and RD designed the study and contributed to the manuscript writing. NR, VS, and AT planned and performed experiments. NR, LL, BW, AT, and RD analyzed the data. All authors contributed to the article and approved the submitted version.

## Funding

This work was supported by a grant from NINDS (NS096148) to RD. The MS brain collection program is supported by NINDS grant R35NS097303 to B. D. Trapp. The Fusion Lumos instrument was purchased *via* an NIH shared instrument grant, 1S10OD023436-01.

## Conflict of Interest

The authors declare that the research was conducted in the absence of any commercial or financial relationships that could be construed as a potential conflict of interest.

## Publisher's Note

All claims expressed in this article are solely those of the authors and do not necessarily represent those of their affiliated organizations, or those of the publisher, the editors and the reviewers. Any product that may be evaluated in this article, or claim that may be made by its manufacturer, is not guaranteed or endorsed by the publisher.

## Glossary

NAGM, Normal appearing gray matter; NAWM, Normal appearing white matter; WML, White matter lesion; GML, Gray matter lesion; LC-MS/MS, Liquid Chromatography with tandem mass spectrometry; LFQ, Label-free quantification; DEPs, Differentially expressed proteins; GO, Gene Ontology; PPI, Protein-protein interaction; MCODE, Molecular Complex Detection; IPA, Ingenuity Pathway Analysis; IHC, Immunohistochemistry; PLP1, Proteolipid Protein 1; MHCII, Major Histocompatibility complex class II; ERMN, Ermin; CNDP1, Carnosine dipeptidase 1; ENPP2, Ectonucleotide Pyrophosphatase/Phosphodiesterase 2; VDAC, Voltage-Dependent Anion Channel; NDUFA5, Ubiquinone Oxidoreductase Subunit A5; ATP5L, ATP Synthase Membrane Subunit G; RHO-A,B,G, Ras homologous protein-family member A,B,G; CLIC4, Chloride intracellular channel protein 4;BIN1, Myc box-dependent-interacting protein 1; CFL2, Cofilin-2; CRK, Adapter molecule crk; FSCN1, Fascin; ANLN, Actin-binding protein anillin;CNTN2, Contactin-2; PLS3, Plastin-3; PFN1, Profilin-1; RAC1, Ras-related C3 botulinum toxin substrate 1; CAP1, Adenylyl cyclase-associated protein 1; PFDN6, Prefoldin subunit 6; CDC42, Cell division control protein 42 homolog; RDX, Radixin; PFDN5, Prefoldin subunit 5; PFDN2, Prefoldin subunit 2; SIRT2, NAD-dependent protein deacetylase sirtuin-2; HINT1, Histidine triad nucleotide-binding protein 1; MAP4, Microtubule-associated protein 4; DPYSL2, Dihydropyrimidinase-related protein 2; STMN1, Stathmin;TPPP: Tubulin polymerization-promoting protein; PEA15, Astrocytic phosphoprotein PEA-15; FNTA, Protein farnesyltransferase/geranylgeranyltransferase type-1 subunit alpha; PAFAH1B1, Platelet-activating factor acetylhydrolase IB subunit alpha; ACTR1A, Alpha-centractin; DCTN2, Dynactin subunit 2; DYNLRB1, Dynein light chain roadblock-type 1;Dynein light chain roadblock-type 2; ARHGDIA, Rho GDP-dissociation inhibitor 1; BCAS1, Breast carcinoma-amplified sequence 1; HAPLN2, Hyaluronan and proteoglycan link protein 2; VCAN, Versican core protein; MAG, Myelin-associated glycoprotein; OMG, Oligodendrocyte-myelin glycoprotein; NDUFV1, NADH dehydrogenase [ubiquinone] flavoprotein 1; UQCRC1, Cytochrome b-c1 complex subunit 1; ATP5F1, ATP synthase F(0) complex subunit B1; ATP5H, ATP synthase subunit d, mitochondrial; ATP5D, ATP synthase subunit delta, mitochondrial; SLC25A4, ADP/ATP translocase 1; SLC25A5, ADP/ATP translocase 2.
