# Supplementary material for: Comparative Proteomic Profiling Identifies Reciprocal Expression of Mitochondrial Proteins Between White and Gray Matter Lesions From Multiple Sclerosis Brains
Source: Front Neurol. 2021 Dec 24;12:779003. doi: 10.3389/fneur.2021.779003 (PMC8740228; doi:10.3389/fneur.2021.779003)
Supplement: Supplementary file 7 [file Table_1.docx]

**Supplementary Table 1:** Cellular source of significantly dysregulated proteins identified in white matter lesions (WMLs) by LC-MS/MS Analysis

| **WMLs Up-regulated proteins** | | | | |
| --- | --- | --- | --- | --- |
| **Protein names** | **Gene names** | **log2(FC)** | **-Log 10 (p)** | **Predicted Cellular Localization** |
| Microtubule-associated protein 2 | MAP2 | 2.8 | 1.5 | Neuron/axon |
| Voltage-dependent anion-selective channel protein 1 | VDAC1 | 2.7 | 3.6 | Neuron/axon |
| Voltage-dependent anion-selective channel protein 2 | VDAC2 | 2.6 | 4.1 | Neuron/axon |
| Synapsin-1 | SYN1 | 2.4 | 2.0 | Neuron/axon |
| Cytochrome b-c1 complex subunit 1, mitochondrial | UQCRC1 | 2.4 | 3.7 | Astrocyte |
| Vesicle-associated membrane protein 2 | VAMP2 | 2.2 | 1.6 | Neuron/axon |
| ADP/ATP translocase 1 | SLC25A4 | 2.2 | 3.5 | Neuron/axon |
| Cytochrome c oxidase subunit 2 | MT-CO2 | 2.1 | 2.1 | Microglia/macrophage |
| Cytochrome b-c1 complex subunit 2, mitochondrial | UQCRC2 | 2.1 | 4.1 | Neuron |
| ProSAAS | PCSK1N | 2.1 | 1.3 | Oligodendrocyte |
| ADP/ATP translocase 2 | SLC25A5 | 2.0 | 2.7 | Astrocyte |
| ATP synthase subunit O, mitochondrial | ATP5O | 2.0 | 3.2 | Neuron |
| NADH-ubiquinone oxidoreductase 75 kDa subunit, mitochondrial | NDUFS1 | 1.9 | 3.2 | Neuron>astrocyte |
| Hexokinase-1 | HK1 | 1.9 | 3.2 | Neuron |
| Neutral amino acid transporter A | SLC1A4 | 1.8 | 1.7 | Astrocyte |
| Amine oxidase [flavin-containing] B | MAOB | 1.8 | 2.2 | Astrocyte |
| ATP synthase subunit g, mitochondrial | ATP5L | 1.8 | 2.9 | Neuron>Astrocyte=Oligodendrocyte |
| Plasma membrane calcium-transporting ATPase 1 | ATP2B1 | 1.7 | 2.6 | Neuron |
| ATP synthase F(0) complex subunit B1, mitochondrial | ATP5F1 | 1.7 | 3.0 | Neuron |
| Calcium/calmodulin-dependent 3,5-cyclic nucleotide phosphodiesterase 1B | PDE1B | 1.7 | 1.6 | Neuron |
| Apolipoprotein E | APOE | 1.7 | 3.2 | Astrocyte |
| Excitatory amino acid transporter 2 | SLC1A2 | 1.7 | 1.4 | Astrocyte |
| ATP synthase subunit alpha, mitochondrial | ATP5A1 | 1.6 | 3.0 | Neuron |
| Ras-related protein Rab-3A | RAB3A | 1.6 | 1.4 | Neuron |
| Neuronal-specific septin-3 | SEPTIN3 | 1.6 | 3.1 | Neuron |
| Sarcoplasmic/endoplasmic reticulum calcium ATPase 2 | ATP2A2 | 1.5 | 2.5 | Neuron |
| Excitatory amino acid transporter 1 | SLC1A3 | 1.5 | 4.2 | Astrocyte |
| ATP synthase subunit gamma, mitochondrial | ATP5C1 | 1.4 | 2.5 | Neuron |
| Calcium/calmodulin-dependent protein kinase type II subunit alpha | CAMK2A | 1.4 | 1.3 | Neuron |
| Beta-soluble NSF attachment protein | NAPB | 1.4 | 2.2 | Neuron |
| Myosin-9 | MYH9 | 1.4 | 1.4 | Endothelial>Macrophage |
| AP-2 complex subunit mu | AP2M1 | 1.4 | 2.4 | Astrocyte>endothelial cell |
| NADH dehydrogenase [ubiquinone] flavoprotein 2, mitochondrial | NDUFV2 | 1.4 | 2.3 | Neuron |
| NADH dehydrogenase [ubiquinone] flavoprotein 1, mitochondrial | NDUFV1 | 1.3 | 3.1 | Astrocyte |
| Syntaxin-1B | STX1B | 1.3 | 2.6 | Neuron |
| Vesicle-fusing ATPase | NSF | 1.3 | 1.9 | Neuron |
| Neuroplastin | NPTN | 1.3 | 1.9 | Neuron |
| Immunoglobulin kappa light chain | IGKC | 1.3 | 1.3 |  |
| AP-2 complex subunit alpha-2 | AP2A2 | 1.3 | 1.6 | Neuron |
| Phenylalanine--tRNA ligase beta subunit | FARSB | 1.3 | 1.5 | Neuron |
| Opioid-binding protein/cell adhesion molecule | OPCML | 1.2 | 2.5 | Neuron |
| Succinate dehydrogenase [ubiquinone] flavoprotein subunit, mitochondrial | SDHA | 1.2 | 3.7 | Astrocyte>Oligodendrocyte |
| ATP synthase subunit d, mitochondrial | ATP5H | 1.2 | 2.3 | Neuron |
| Isocitrate dehydrogenase [NAD] subunit alpha, mitochondrial | IDH3A | 1.2 | 1.7 | Astrocyte |
| ATP synthase subunit beta, mitochondrial | ATP5B | 1.2 | 2.7 | Neuron |
| Nucleoplasmin-2 | NPM2 | 1.2 | 1.4 | Endothelial |
| Protein S100-A13 | S100A13 | 1.1 | 2.6 | Astrocyte |
| NADH dehydrogenase [ubiquinone] 1 alpha subcomplex subunit 5 | NDUFA5 | 1.1 | 1.7 | Neuron |
| AP-2 complex subunit alpha-1 | AP2A1 | 1.1 | 1.7 | Astrocyte>neuron |
| Creatine kinase U-type, mitochondrial | CKMT1A | 1.1 | 2.8 | Neuron |
| Syntaxin-binding protein 1 | STXBP1 | 1.1 | 1.5 | Neuron |
| Glial fibrillary acidic protein | GFAP | 1.1 | 1.3 | Astrocyte |
| ATP synthase subunit delta, mitochondrial | ATP5D | 1.1 | 1.8 | Astrocyte |
| ATP-dependent 6-phosphofructokinase, platelet type | PFKP | 1.0 | 1.7 | Astrocyte |
| Heat shock 70 kDa protein 12A | HSPA12A | 1.0 | 1.5 | Neuron |
| Mitochondrial import receptor subunit TOM70 | TOMM70A | 1.0 | 2.0 | Neuron |
| Dynamin-1-like protein | DNM1L | 1.0 | 1.5 | Neuron |
| ATP-dependent 6-phosphofructokinase, muscle type | PFKM | 1.0 | 2.3 | Astrocyte>neuron |
| 4F2 cell-surface antigen heavy chain | SLC3A2 | 1.0 | 3.4 | Astrocyte |
| Catenin alpha-2 | CTNNA2 | 1.0 | 1.8 | Astrocyte |
| Solute carrier family 2, facilitated glucose transporter member 1 | SLC2A1 | 0.9 | 1.4 | Endothelial>astrocyte |
| Hypoxia up-regulated protein 1 | HYOU1 | 0.9 | 3.0 | Astrocyte |
| Alpha-actinin-1 | ACTN1 | 0.9 | 2.0 | Neuron>endothelial |
| Syntaxin-1A | STX1A | 0.9 | 1.8 | Endothelial |
| Adipocyte plasma membrane-associated protein | APMAP | 0.9 | 2.7 | Microglia/macrophase>neuron |
| Neuronal membrane glycoprotein M6-a | GPM6A | 0.9 | 1.7 | Astrocyte>Neuron |
| Endophilin-B2 | SH3GLB2 | 0.9 | 1.6 | endothelial |
| Voltage-dependent calcium channel subunit alpha-2/delta-1 | CACNA2D1 | 0.9 | 1.4 | Neuron |
| Cytoplasmic FMR1-interacting protein 2 | CYFIP2 | 0.9 | 1.9 | Neuron |
| EH domain-containing protein 3 | EHD3 | 0.9 | 1.3 | Neuron>Oligodendrocyte |
| Synaptosomal-associated protein 25 | SNAP25 | 0.9 | 2.0 | Neuron |
| Heat shock protein beta-1 | HSPB1 | 0.9 | 1.4 | Astrocyte>endothelial cell |
| Cytochrome c | CYCS | 0.9 | 2.1 | Neuron |
| Tubulin beta-3 chain | TUBB3 | 0.9 | 1.6 | Neuron=astrocyte |
| CD99 antigen-like protein 2 | CD99L2 | 0.9 | 3.2 | microglia/macrophage |
| Annexin A7 | ANXA7 | 0.9 | 2.2 | All As=Neu=mic=endo |
| Guanine nucleotide-binding protein G(o) subunit alpha | GNAO1 | 0.8 | 2.0 | Astrocyte=Neuron |
| Adenylyl cyclase-associated protein 2 | CAP2 | 0.8 | 1.8 | Neuron |
| Sodium/potassium-transporting ATPase subunit alpha-2 | ATP1A2 | 0.8 | 2.0 | Astrocyte |
| Endophilin-A1 | SH3GL2 | 0.8 | 1.7 | Neuron |
| Sorcin | SRI | 0.8 | 1.6 | Astrocyte |
| Neurochondrin | NCDN | 0.8 | 1.5 | Neuron |
| Septin-11 | SEPT11 | 0.8 | 3.0 | NA |
| Cullin-associated NEDD8-dissociated protein 1 | CAND1 | 0.8 | 2.7 | Neuron |
| Annexin A11 | ANXA11 | 0.8 | 1.9 | Endotheliam>microglia/macrophage |
| Sodium/potassium-transporting ATPase subunit beta-1 | ATP1B1 | 0.8 | 2.4 | Neuron |
| NADH-cytochrome b5 reductase 3 | CYB5R3 | 0.8 | 2.3 | endothelial>astrocyte |
| Clathrin coat assembly protein AP180 | SNAP91 | 0.8 | 1.7 | Neuron |
| Sodium/potassium-transporting ATPase subunit beta-2 | ATP1B2 | 0.8 | 1.7 | Astrocyte |
| Gamma-soluble NSF attachment protein | NAPG | 0.8 | 1.8 | Neuron |
| Ankyrin-2 | ANK2 | 0.8 | 2.0 | Neuron>Astrocyte |
| Nucleoside diphosphate kinase B | NME2 | 0.7 | 3.2 | Neuron>Microglia/macrophage |
| Hepatocyte cell adhesion molecule | HEPACAM | 0.7 | 1.6 | Astrocyte |
| Sodium/potassium-transporting ATPase subunit alpha-3 | ATP1A3 | 0.7 | 1.6 | Neuron |
| Dihydrolipoyllysine-residue acetyltransferase component of pyruvate dehydrogenase complex, mitochondrial | DLAT | 0.7 | 1.3 | Neuron |
| Citrate synthase, mitochondrial | CS | 0.7 | 1.7 | Neuron=astrocyte |
| Sodium/potassium-transporting ATPase subunit alpha-1 | ATP1A1 | 0.7 | 1.9 | Neuron |
| Proline-rich transmembrane protein 2 | PRRT2 | 0.7 | 1.4 | Astrocyte |
| Elongation factor 1-alpha 2 | EEF1A2 | 0.7 | 1.4 | Neuron |
| AP-2 complex subunit beta | AP2B1 | 0.7 | 1.4 | Neuron |
| Tyrosine-protein phosphatase non-receptor type substrate 1 | SIRPA | 0.6 | 1.5 | astrocyte>microglia/macrophage |
| Transcriptional activator protein Pur-alpha | PURA | 0.6 | 1.5 | Oligodendrocyte>Neuron |
| Clusterin | CLU | 0.6 | 2.6 | Astrocyte |
| Dihydrolipoyllysine-residue succinyltransferase component of 2-oxoglutarate dehydrogenase complex, mitochondrial | DLST | 0.6 | 1.5 | Neuron>Microglia/macrophage |
| Alpha-soluble NSF attachment protein | NAPA | 0.6 | 1.4 | Astrocyte>endothelial cell |
| **WMLs Downregulated proteins** | | | | |
| Ermin | ERMN | -2.4 | 2.4 | Oligodendrocyte |
| Versican core protein | VCAN | -2.2 | 3.5 | Oligodendrocyte |
| Hyaluronan and proteoglycan link protein 2 | HAPLN2 | -2.1 | 2.5 | Oligodendrocyte |
| NADH-cytochrome b5 reductase 2 | CYB5R2 | -2.1 | 4.2 | Oligodendrocyte |
| Ectonucleotide pyrophosphatase/phosphodiesterase family member 2 | ENPP2 | -2.1 | 1.3 | Oligodendrocyte |
| Breast carcinoma-amplified sequence 1 | BCAS1 | -1.9 | 3.0 | Oligodendrocyte |
| Myelin-associated glycoprotein | MAG | -1.9 | 3.4 | Oligodendrocyte |
| Ectonucleotide pyrophosphatase/phosphodiesterase family member 6 | ENPP6 | -1.9 | 3.0 | Oligodendrocyte |
| Chloride intracellular channel protein 4 | CLIC4 | -1.8 | 1.6 | Oligodendrocyte>endothelial |
| Galectin-3-binding protein | LGALS3BP | -1.8 | 2.4 | Astrocytes |
| Protein S100-B | S100B | -1.7 | 1.6 | Astrocyte |
| Rho-related GTP-binding protein RhoG | RHOG | -1.7 | 1.8 | Microglia/macrophage> Oligodendrocyte |
| Ribonuclease inhibitor | RNH1 | -1.7 | 3.1 | Astrocyte |
| Myc box-dependent-interacting protein 1 | BIN1 | -1.6 | 3.0 | Microglia/macrophage |
| Lysosome-associated membrane glycoprotein 2 | LAMP2 | -1.6 | 3.3 | Oligodendrocyte |
| Serine/threonine-protein phosphatase CPPED1 | CPPED1 | -1.6 | 3.2 | Oligodendrocyte |
| Perilipin-3 | PLIN3 | -1.5 | 3.2 | Astrocyte |
| Phosphatidylinositol 5-phosphate 4-kinase type-2 alpha | PIP4K2A | -1.5 | 4.0 | Oligodendrocyte |
| Oligodendrocyte-myelin glycoprotein | OMG | -1.5 | 2.2 | Oligodendrocyte>astrocyte |
| Cysteine and glycine-rich protein 1 | CSRP1 | -1.5 | 2.1 | Astrocyte>oligodendrocyte |
| Transaldolase | TALDO1 | -1.5 | 2.8 | Oligodendrocyte>macrophage/microglia |
| Transforming protein RhoA | RHOA | -1.4 | 2.7 | Microglia/macrophage>oligodendrocyte |
| Dihydropyrimidinase-related protein 5 | DPYSL5 | -1.4 | 2.3 | Neuron |
| Signal transducing adapter molecule 1 | STAM | -1.4 | 3.2 | Neuron |
| Cofilin-2 | CFL2 | -1.4 | 2.2 | Oligodendrocyte |
| Heat shock-related 70 kDa protein 2 | HSPA2 | -1.3 | 5.3 | Oligodendrocyte |
| Heme-binding protein 1 | HEBP1 | -1.3 | 4.8 | Oligodendrocyte |
| Serine/arginine-rich splicing factor 3 | SRSF3 | -1.3 | 2.0 | Microglia/macrophage=Neuron |
| Acidic leucine-rich nuclear phosphoprotein 32 family member A | ANP32A | -1.3 | 3.2 | Neuron=Oligodendrocyte |
| Serotransferrin | TF | -1.3 | 2.1 | Oligodendrocyte |
| Glycolipid transfer protein | GLTP | -1.3 | 1.8 | Oligodendrocyte |
| Dihydropteridine reductase | QDPR | -1.3 | 2.5 | Oligodendrocyte |
| Protein CutA | CUTA | -1.3 | 1.7 | Neuron |
| Stathmin | STMN1 | -1.2 | 2.4 | Neuron>Oligodendrocyte |
| Gelsolin | GSN | -1.2 | 2.9 | Microglia/macrophage>Astrocyte |
| Protein farnesyltransferase/geranylgeranyltransferase type-1 subunit alpha | FNTA | -1.2 | 1.8 | Oligodendrocyte |
| Kynurenine--oxoglutarate transaminase 1 | CCBL1 | -1.2 | 1.3 | Astrocyte>Neuron |
| Carbonic anhydrase 2 | CA2 | -1.2 | 2.5 | Oligodendrocyte |
| Adapter molecule crk | CRK | -1.2 | 2.8 | Astrocyte |
| Brevican core protein | BCAN | -1.2 | 2.3 | Astrocyte>oligodendrocyte |
| Ras-related C3 botulinum toxin substrate 1 | RAC1 | -1.2 | 1.7 | Oligodendrocyte>Neuron |
| Fascin | FSCN1 | -1.1 | 4.2 | Endothelial |
| Delta-aminolevulinic acid dehydratase | ALAD | -1.1 | 1.9 | Oligodendrocyte |
| Plastin-3 | PLS3 | -1.1 | 2.6 | Neuron |
| NAD-dependent protein deacetylase sirtuin-2 | SIRT2 | -1.1 | 1.6 | Oligodendrocyte |
| Ganglioside GM2 activator | GM2A | -1.1 | 2.4 | Microglia/macrophage>astrocyte |
| Nucleolin | NCL | -1.1 | 1.8 | Oligodendrocyte |
| Fibroblast growth factor 1 | FGF1 | -1.1 | 2.0 | Oligodendrocyte |
| Protein LZIC | LZIC | -1.1 | 1.7 | Oligodendrocyte |
| Ras-related protein Rab-21 | RAB21 | -1.1 | 2.9 | Oligodendrocyte |
| CD9 antigen | CD9 | -1.0 | 1.9 | Oligodendrocyte |
| Actin-binding protein anillin | ANLN | -1.0 | 1.3 | Oligodendrocyte |
| Nuclear transport factor 2 | NUTF2 | -1.0 | 1.4 | Neuron |
| Superoxide dismutase [Cu-Zn] | SOD1 | -1.0 | 2.2 | Neuron |
| Cytosolic non-specific dipeptidase | CNDP1 | -1.0 | 2.6 | Oligodendrocyte |
| Phospholysine phosphohistidine inorganic pyrophosphate phosphatase | LHPP | -1.0 | 1.8 | Astrocyte>Oligodendrocyte |
| Retinal dehydrogenase 1 | ALDH1A1 | -1.0 | 1.6 | Astrocyte |
| Tubulin polymerization-promoting protein | TPPP | -0.9 | 2.3 | Oligodendrocyte |
| Cell adhesion molecule 4 | CADM4 | -0.9 | 3.0 | Oligodendrocyte |
| Glucosamine-6-phosphate isomerase 1 | GNPDA1 | -0.9 | 1.7 | Macrophage/microglia |
| Ferritin heavy chain | FTH1 | -0.9 | 1.3 | Macrophage/microglia>Oligodendrocyte |
| Prosaposin | PSAP | -0.9 | 2.0 | Astrocyte |
| Crk-like protein | CRKL | -0.9 | 2.9 | Astrocyte |
| Cell division control protein 42 homolog | CDC42 | -0.9 | 2.3 | Neuron |
| Rho-related GTP-binding protein RhoB | RHOB | -0.9 | 2.2 | Endothelial |
| Microtubule-associated protein 4 | MAP4 | -0.9 | 2.2 | Oligodendrocyte |
| Tenascin | TNC | -0.9 | 1.7 | Astrocyte |
| Hsc70-interacting protein | ST13 | -0.9 | 2.0 | Oligodendrocyte |
| Prefoldin subunit 5 | PFDN5 | -0.9 | 2.5 | Neuron |
| Serpin B6 | SERPINB6 | -0.9 | 1.3 | Endothelial |
| Contactin-2 | CNTN2 | -0.9 | 2.2 | Oligodendrocyte |
| UV excision repair protein RAD23 homolog B | RAD23B | -0.9 | 2.3 | Neuron |
| Peptidyl-prolyl cis-trans isomerase A | PPIA | -0.9 | 3.2 | Neuron |
| Tripartite motif-containing protein 2 | TRIM2 | -0.9 | 2.6 | Oligodendrocyte |
| Ras-related protein Rap-1A | RAP1A | -0.9 | 2.0 | Microglia/macrophage |
| 14-3-3 protein theta | YWHAQ | -0.9 | 2.3 | Oligodendrocyte |
| Carbonyl reductase [NADPH] 1 | CBR1 | -0.9 | 1.5 | Oligodendrocyte |
| Endonuclease domain-containing 1 protein | ENDOD1 | -0.9 | 1.3 | Oligodendrocyte |
| Enolase-phosphatase E1 | ENOPH1 | -0.8 | 2.8 | Oligodendrocyte>Neuron |
| Dynein light chain roadblock-type 1;Dynein light chain roadblock-type 2 | DYNLRB1 | -0.8 | 1.3 | Neuron |
| Dihydropyrimidinase-related protein 2 | DPYSL2 | -0.8 | 3.0 | Astrocyte>Oligodendrocyte |
| Prefoldin subunit 6 | PFDN6 | -0.8 | 2.0 | Neuron |
| S-formylglutathione hydrolase | ESD | -0.8 | 1.7 | Neuron |
| Acetyl-CoA acetyltransferase, cytosolic | ACAT2 | -0.8 | 2.1 | Neuron |
| Histidine triad nucleotide-binding protein 1 | HINT1 | -0.8 | 2.9 | Neuron |
| Profilin-1 | PFN1 | -0.8 | 2.1 | Microglia/macrophage |
| Isoamyl acetate-hydrolyzing esterase 1 homolog | IAH1 | -0.8 | 1.5 | Neuron |
| Platelet-activating factor acetylhydrolase IB subunit alpha | PAFAH1B1 | -0.8 | 1.7 | Neuron |
| Prefoldin subunit 2 | PFDN2 | -0.8 | 3.0 | Neuron=Oligodendrocyte |
| Alpha-centractin | ACTR1A | -0.8 | 2.9 | Astrocyte |
| Secernin-1 | SCRN1 | -0.7 | 3.0 | Neuron |
| Glycine amidinotransferase, mitochondrial | GATM | -0.7 | 1.8 | Astrocyte>macrophage/microglia>oligodendrocyte |
| Protein SET | SET | -0.7 | 1.7 | Neuron |
| Rho GDP-dissociation inhibitor 1 | ARHGDIA | -0.7 | 2.6 | Microglia/macrophage |
| Adenylyl cyclase-associated protein 1 | CAP1 | -0.7 | 2.2 | Microglia/macrophage |
| Heterogeneous nuclear ribonucleoprotein D0 | HNRNPD | -0.7 | 2.3 | Macrophage/microglia |
| Dynactin subunit 2 | DCTN2 | -0.7 | 1.7 | Neuron |
| Prolyl endopeptidase | PREP | -0.7 | 1.3 | Neuron |
| Astrocytic phosphoprotein PEA-15 | PEA15 | -0.7 | 1.5 | Astrocyte |
| Ubiquitin-conjugating enzyme E2 L3 | UBE2L3 | -0.7 | 1.9 | Astrocyte |
| Adenosylhomocysteinase | AHCY | -0.7 | 2.0 | Neuron |
| Transketolase | TKT | -0.7 | 1.4 | Astrocyte |
| N(G),N(G)-dimethylarginine dimethylaminohydrolase 2 | DDAH2 | -0.7 | 1.6 | Macrophage/microglia |
| Clathrin light chain A | CLTA | -0.7 | 1.4 | Neuron>Oligodendrocyte |
| Protein FAM49B | FAM49B | -0.6 | 1.3 | Macrophage/microglia>Neuron |
| CD166 antigen | ALCAM | -0.6 | 2.4 | Oligodendrocye>Neuron |
| Phosphatidylethanolamine-binding protein 1 | PEBP1 | -0.6 | 1.7 | Neuron |
| Polyadenylate-binding protein 1 | PABPC1 | -0.6 | 1.3 | Macrophage/microglia |
| Ubiquitin carboxyl-terminal hydrolase isozyme L1 | UCHL1 | -0.6 | 1.6 | Neuron |
| Poly(rC)-binding protein 1 | PCBP1 | -0.6 | 1.9 | Neuron=microglia/macrophae |
| Radixin | RDX | -0.6 | 1.8 | Oligodendrocyte |
| Signal recognition particle 14 kDa protein | SRP14 | -0.6 | 1.3 | Neuron |
| Methionine adenosyltransferase 2 subunit beta | MAT2B | -0.6 | 1.4 | Neuron |
| Tenascin-R | TNR | -0.6 | 2.6 | Oligodendrocyte |
| Triosephosphate isomerase | TPI1 | -0.6 | 2.1 | Neuron |
| Serine/threonine-protein phosphatase PP1-beta catalytic subunit | PPP1CB | -0.6 | 1.4 | Neuron |
| Ras-related protein Ral-A | RALA | -0.6 | 2.3 | Oligodendrocyte |
| Lactoylglutathione lyase | GLO1 | -0.6 | 1.5 | Astrocyte |
| Glyoxylate reductase/hydroxypyruvate reductase | GRHPR | -0.6 | 1.6 | Macrophage/microglia |
