# Supplementary material for: Comparative Proteomic Profiling Identifies Reciprocal Expression of Mitochondrial Proteins Between White and Gray Matter Lesions From Multiple Sclerosis Brains
Source: Front Neurol. 2021 Dec 24;12:779003. doi: 10.3389/fneur.2021.779003 (PMC8740228; doi:10.3389/fneur.2021.779003)
Supplement: Supplementary file 8 [file Table_2.docx]

**Supplementary Table 2:** Cellular source of significantly dysregulated proteins identified in Gray matter (GMLs) by LCMS-MS Analysis

| **GMLs Up-regulated proteins** | | | | |
| --- | --- | --- | --- | --- |
| **Protein names** | **Gene names** | **log2FC** | **-Log 10 (p)** | **Predicted Cellular Localization** |
| Cytosolic 10-formyltetrahydrofolate dehydrogenase | ALDH1L1 | 0.9 | 1.3 | Astrocyte |
| Mannose-6-phosphate isomerase | MPI | 0.7 | 2.8 | Neuron>astrocyte |
| Astrocytic phosphoprotein PEA-15 | PEA15 | 0.7 | 1.8 | Astrocyte |
| Apolipoprotein A-I | APOA1 | 0.7 | 1.9 | Endothelial |
| Cysteine-rich protein 2 | CRIP2 | 0.7 | 1.3 | Endothelial |
| Tyrosine-protein phosphatase non-receptor type 11 | PTPN11 | 0.7 | 3.7 | Oligodendrocyte |
| Plectin | PLEC | 0.6 | 1.4 | Astrocyte |
| MAP7 domain-containing protein 1 | MAP7D1 | 0.6 | 1.8 | Oligodendrocyte |
| Dihydropyrimidinase-related protein 4 | DPYSL4 | 0.6 | 1.6 | Neuron |
| Inositol polyphosphate 1-phosphatase | INPP1 | 0.6 | 2.8 | Oligodendrocyte |
| Biliverdin reductase A | BLVRA | 0.6 | 1.3 | Neuron |
| Glucose 1,6-bisphosphate synthase | PGM2L1 | 0.6 | 2.1 | Neuron |
| 26S protease regulatory subunit 6A | PSMC3 | 0.6 | 1.3 | Endothelial |
| 3-ketoacyl-CoA thiolase, peroxisomal | ACAA1 | 0.6 | 1.8 | Astrocyte |
| 3-hydroxybutyrate dehydrogenase type 2 | BDH2 | 0.6 | 1.3 | Astrocyte |
| Glutathione S-transferase Mu 3 | GSTM3 | 0.6 | 1.3 | Astrocyte |
| Coronin-1C | CORO1C | 0.6 | 1.5 | Oligodendrocyte |
| Four and a half LIM domains protein 1 | FHL1 | 0.6 | 1.3 | Neuron |
| Fermitin family homolog 2 | FERMT2 | 0.6 | 1.9 | Astrocyte |
| **GMLs downregulated proteins** | | | | |
| Solute carrier family 12 member 5 | SLC12A5 | -1.2 | 1.5 | Neuron (exclusive) |
| ATP synthase-coupling factor 6, mitochondrial | ATP5J | -1.0 | 2.8 | Neuron |
| Potassium voltage-gated channel subfamily A member 2 | KCNA2 | -1.0 | 1.7 | Astrocyte>Neuron |
| Cytochrome b-c1 complex subunit 6, mitochondrial | UQCRH | -0.9 | 2.1 | Neuron |
| NADH dehydrogenase [ubiquinone] 1 alpha subcomplex subunit 7 | NDUFA7 | -0.9 | 2.0 | Neuron=astrocyte |
| Sodium/potassium-transporting ATPase subunit beta-2 | ATP1B2 | -0.9 | 1.6 | Astrocyte |
| Cytochrome c oxidase subunit 5A, mitochondrial | COX5A | -0.8 | 3.4 | Neuron |
| Hyaluronan and proteoglycan link protein 4 | HAPLN4 | -0.8 | 1.6 | Neuron |
| CDGSH iron-sulfur domain-containing protein 1 | CISD1 | -0.8 | 1.7 | Neuron>Astrocyte |
| ATP synthase subunit delta, mitochondrial | ATP5D | -0.8 | 2.7 | Astrocyte>oligodendrocyte |
| ATP synthase F(0) complex subunit B1, mitochondrial | ATP5F1 | -0.8 | 1.7 | Neuron |
| Up-regulated during skeletal muscle growth protein 5 | USMG5 | -0.8 | 1.9 | Neuron |
| Sideroflexin-1 | SFXN1 | -0.8 | 1.6 | Neuron |
| ATP synthase subunit g, mitochondrial | ATP5L | -0.8 | 1.7 | Neuron |
| ADP/ATP translocase 3 | SLC25A6 | -0.8 | 1.7 | Endothelial>microglia/macrophage |
| Cytochrome c oxidase subunit 6B1 | COX6B1 | -0.7 | 2.6 | Neuron |
| Prenylcysteine oxidase 1 | PCYOX1 | -0.7 | 1.7 | Neuron>Astrocyte |
| Cytochrome b-c1 complex subunit 7 | UQCRB | -0.7 | 2.0 | Neuron |
| ADP/ATP translocase 1 | SLC25A4 | -0.7 | 1.6 | Neuron |
| Cytochrome b-c1 complex subunit 8 | UQCRQ | -0.7 | 1.5 | Neuron>Oligodendrocyte |
| Cytochrome c oxidase subunit NDUFA4 | NDUFA4 | -0.7 | 1.5 | Neuron |
| Cell cycle exit and neuronal differentiation protein 1 | CEND1 | -0.7 | 1.9 | Astrocyte |
| Cytochrome b-c1 complex subunit 1, mitochondrial | UQCRC1 | -0.7 | 1.9 | Astrocyte |
| Phosphate carrier protein, mitochondrial | SLC25A3 | -0.7 | 1.3 | microglia/macrophage |
| NADH dehydrogenase [ubiquinone] 1 alpha subcomplex subunit 5 | NDUFA5 | -0.7 | 1.8 | Neuron |
| Calcium-binding mitochondrial carrier protein Aralar1 | SLC25A12 | -0.7 | 1.4 | Neuron |
| ADP/ATP translocase 2 | SLC25A5 | -0.7 | 1.3 | Astrocyte |
| Cytochrome c oxidase subunit 4 isoform 1, mitochondrial | COX4I1 | -0.7 | 1.8 | Endothelial>macrophage/microglia |
| Cytochrome b-c1 complex subunit Rieske, mitochondrial | UQCRFS1 | -0.6 | 1.9 | Neuron |
| NADH dehydrogenase [ubiquinone] flavoprotein 1, mitochondrial | NDUFV1 | -0.6 | 2.1 | Astrocyte |
| Excitatory amino acid transporter 2 | SLC1A2 | -0.6 | 1.8 | Astrocyte |
| ATP synthase subunit d, mitochondrial | ATP5H | -0.6 | 1.7 | Neuron |
| Sodium- and chloride-dependent GABA transporter 1 | SLC6A1 | -0.6 | 2.6 | Neuron>astrocyte |
| NADH dehydrogenase [ubiquinone] 1 alpha subcomplex subunit 8 | NDUFA8 | -0.6 | 1.8 | Neuron |
| Endonuclease domain-containing 1 protein | ENDOD1 | -0.6 | 1.4 | Oligodendrocyte>astrocyte |
| Guanine nucleotide-binding protein G(I)/G(S)/G(O) subunit gamma-3 | GNG3 | -0.6 | 2.0 | Neuron |
| NADH dehydrogenase [ubiquinone] iron-sulfur protein 5 | NDUFS5 | -0.6 | 1.9 | Neuron |
| Disintegrin and metalloproteinase domain-containing protein 22 | ADAM22 | -0.6 | 1.7 | Neuron |
| 28S ribosomal protein S36, mitochondrial | MRPS36 | -0.6 | 1.5 | Neuron |
| Cytochrome c oxidase subunit 5B, mitochondrial | COX5B | -0.6 | 2.0 | Astrocyte |
